# Comparison of visualization tools for single-cell RNAseq data

**DOI:** 10.1093/nargab/lqaa052

**Published:** 2020-07-29

**Authors:** Batuhan Cakir, Martin Prete, Ni Huang, Stijn van Dongen, Pinar Pir, Vladimir Yu Kiselev

**Affiliations:** Wellcome Sanger Institute, Hinxton, CB10 1SA, UK; Gebze Technical University, Department of Bioengineering, Gebze, Kocaeli, 41400, Turkey; Wellcome Sanger Institute, Hinxton, CB10 1SA, UK; Wellcome Sanger Institute, Hinxton, CB10 1SA, UK; Wellcome Sanger Institute, Hinxton, CB10 1SA, UK; Gebze Technical University, Department of Bioengineering, Gebze, Kocaeli, 41400, Turkey; Wellcome Sanger Institute, Hinxton, CB10 1SA, UK

## Abstract

In the last decade, single cell RNAseq (scRNAseq) datasets have grown in size from a single cell to millions of cells. Due to its high dimensionality, it is not always feasible to visualize scRNAseq data and share it in a scientific report or an article publication format. Recently, many interactive analysis and visualization tools have been developed to address this issue and facilitate knowledge transfer in the scientific community. In this study, we review several of the currently available scRNAseq visualization tools and benchmark the subset that allows to visualize the data on the web and share it with others. We consider the memory and time required to prepare datasets for sharing as the number of cells increases, and additionally review the user experience and features available in the web interface. To address the problem of format compatibility we have also developed a user-friendly R package, *sceasy*, which allows users to convert their own scRNAseq datasets into a specific data format for visualization.

## INTRODUCTION

In just a decade, the number of cells profiled in each scRNAseq experiment has increased from ∼1000 cells to millions of cells (1), thanks to the advent of sequencing protocols, from well-based (2–4) to droplet-based (5,6) and the ever-decreasing cost of sequencing. In parallel, many computational methods have been developed to analyse and quantify scRNAseq data (7–12). A typical scRNAseq analysis pipeline starts from the raw reads, which are processed to create an expression matrix, containing the expression values of every gene in every cell. Further downstream analysis is then performed where cells are clustered and the cluster-specific marker genes are identified to annotate cells with corresponding cell types. The results are then visualized using non-linear embedding methods, such as tSNE (13) or UMAP (14) usually in a two-dimensional (2D) space where each cell gets a pair of X-Y coordinates defining its position on the visualization plot. Finally, the visualizations are used to assess the obtained cell types by highlighting the cell metadata (information about cells in a given experiment, e.g. batch, donor etc.) or the expression of specific genes across the cell types. This assessment can only be performed in an interactive manner. However, when the results are shared as a report or published in a paper format (a static 2D image), it is only possible to see a snapshot of the analysis corresponding to a single gene and a single set of cell metadata. Recently the ability to analyze, visualize the data in an interactive way has attracted a lot of attention and advances in web technologies have led to the development of multiple tools for sharing the analysis results via a web interface.

In this paper we attempt to give an overview of a number of currently available tools to help researchers choose an approach for data visualization. In the broader landscape of scRNAseq visualization we initially consider 13 popular interactive analysis and visualization tools and give an overview of their features. We then select those tools that provide web sharing functionality and benchmark them against each other by means of their performance on datasets of different sizes (from 5000 to 2 million cells). We also evaluated user experience (UX) features of these tools. Finally, since all of the tools have different input requirements, we developed an R package, *sceasy*, for flexible conversion of one data format to another (see ‘Materials and Methods’ section).

## MATERIALS AND METHODS

### Datasets

For benchmarking we utilized a mouse embryo development scRNAseq dataset with accession number GSE119945 (15) containing 2.07 million cells with cell and gene metadata and including precalculated cluster assignments and tSNE coordinates. The data and metadata were converted from the input text format to an AnnData object in scanpy. This object was then subsampled in scanpy to generate datasets of different sizes (5 000, 10 000, 25 000, 50 000, 100 000, 250 000, 500 000, 1 000 000, 1 500 000 and 2 000 000 cells) used for performance benchmarking.

### Profiling

Benchmarking tests were done on a virtual Ubuntu OS 16.04 with 23GB of RAM and 2GHz Intel Xeon Processor with 16 cores.

iSEE and scSVA are both R packages and therefore were tested by using *profvis*, a package for profiling R scripts (16). The highest value of the *‘memalloc’* slot with the label of *‘shiny::runApp’* was considered as preprocessing memory, and the last value in the *‘time’* slot was considered as preprocessing time.

For all the other tools (except cellxgene) the characteristics were measured by running Linux command /usr/bin/time -v, and using ‘*Maximum resident set size (kbytes)’* output for RAM usage and using the sum of *‘User time (seconds)’* and *‘System time (seconds)’* outputs for preprocessing times. For cellxgene the gnomon command was used with elapsed-total option to measure the preprocessing times. For SCope the preprocessing time was profiled only from the server side (the timed process was scope-server). The preprocessing times included both the internal data import time (only for UCSC Cell Browser and Single Cell Explorer) and server start-up time. Both UCSC Cell Browser and Single Cell Explorer spent a considerable amount of time on the data import as they need to convert the data into other structures (json files and MongoDB database, respectively) before being able to visualize it. For UCSC Cell Browser, scanpy's (17) scanpy.external.exporting.cellbrowser was used to perform the conversion. For Single Cell Explorer, ProcessPipline.insertToDB function from the scpipline.py library provided by the authors was used.

### Data format conversion

We developed the R package sceasy for converting data formats frequently used in scRNA-seq analysis, namely Seurat object, SingleCellExperiment (SCE) object, Loom object, to AnnData object. It also supports conversion from Seurat object to SCE object, and between SCE and Loom objects. The package complements existing conversion functions such as those in Seurat and scran. Examples of the conversion functions can be found at the sceasy GitHub page: https://github.com/cellgeni/sceasy

## RESULTS

### Tools overview

We considered 13 popular scRNAseq analysis and visualization tools: ASAP (18), BioTuring Single Cell Browser (Bbrowser) (19), cellxgene (20), Granatum (21), iSEE (22), loom-viewer (23), Loupe Cell Browser (24), SCope (25), scSVA (26), scVI (27), Single Cell Explorer (28), SPRING (29) and UCSC Cell Browser (30). Table 1 compares these tools in terms of cloud and web support, containerization, supported input formats and developer activity.

**Table 1.** Overview of the visualization tools and their capabilities

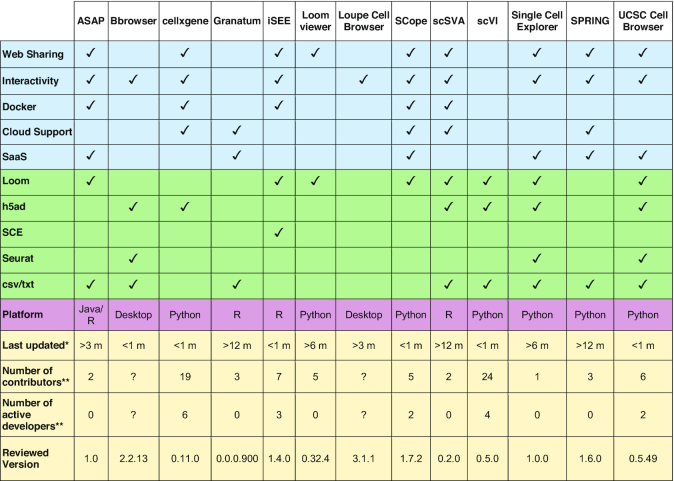

*Latest GitHub commit (checked on 9 June 2020), **checked at submission time. Web Sharing corresponds to the ability of hosting and sharing a web page with data visualization. Interactivity corresponds to the ability of exploring the data in an interactive way as opposed to static images. Docker indicates whether a docker image with the tool is provided by the developers. Cloud Support indicates whether the authors provided instructions on how to deploy their web interface on a public cloud. SaaS (Software as a Service) corresponds to availability of a hosted online version of the tool provided by the authors which only requires uploading of input files by the user. The rows marked Loom, h5ad, SCE (SingleCellExperiment), Seurat, csv/txt indicate the different standard input data formats that are in use.

### Input formats

The tools vary in the ability to use different input file/data formats (green color in Table 1). We focused on input formats supported out of the box. The csv/txt format is the most commonly accepted one and is supported by eight tools. More specialized formats such as h5ad and loom are accepted by six tools. R-based SingleCellExperiment (SCE) and Seurat are accepted by one and three tools, respectively. To make it possible for the users to visualize their datasets in different ways we have developed the *sceasy* R package for file format conversion (see ‘Materials and Methods’ section), which is available on Github at https://github.com/cellgeni/sceasy.

### Data hosting, publishing and sharing via the web

These features are indicated in blue in Table 1. *Web Sharing* corresponds to the ability of hosting and sharing a web page with data visualization, whereas the *SaaS* feature corresponds to availability of a hosted online version of the tool provided by the authors which only requires uploading of input files by the user. An example is ASAP which is a comprehensive hosting platform at École Polytechnique Fédérale de Lausanne (EPFL) and as such it does not require a local or cloud installation. *Cloud Support* indicates whether the authors provided tools and instructions on how to deploy their web interface on a public cloud.

### Selection of tools for web sharing

For further comparison we selected actively developed (updated during the last 6 months) tools that can be used for web sharing. For ASAP we were not able to host it locally with the provided Docker image, hence it was excluded from the comparison. SPRING was excluded from the comparison because it was not updated recently and in addition no Docker image is provided.

### Web sharing steps

For the end user to see a visualization web page it usually requires three steps to be completed: the input files have to be prepared (e.g. to create a database or to convert to another data format, different from the ones in Table 1), the back-end server has to be started and, finally, the web page should be served to the user in their web browser. For all of the considered tools the memory and time needed for the latter step are negligible compared to the first two steps. We defined the maximum memory and total time required for the first two steps as the preprocessing memory (RAM) and the preprocessing time and measured how they depend on the number of cells in the input data. All of the tools were run with the default parameters. iSEE was run for both SCE and loom input formats.

### Summary of benchmarking results

**Figure 1.**
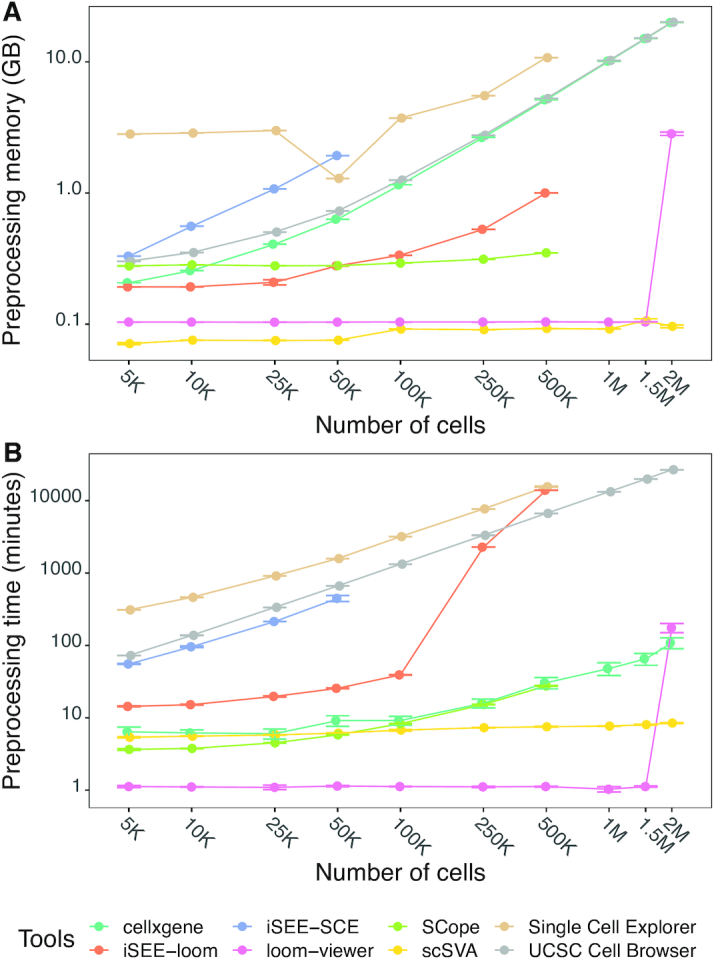
Preprocessing RAM usage (**A**) and preprocessing times (**B**) of the visualization tools. The points on the plots represent mean values and error bars represent standard error across five independent runs. Preprocessing times include input preparation and starting of the back-end server.

Figure 1 summarizes the benchmarking results. iSEE-loom, SCope, scSVA and loom-viewer all enable efficient integration with the hierarchical data format (HDF5) from which loom and h5ad formats are derived. HDF5 format allows for on-demand loading, i.e. the necessary data is only loaded in RAM when it is needed by the application. In this case, at the start the tools only use the coordinates of the cells without loading other input data into memory. scSVA and loom-viewer are the most efficient HDF5-backed tools with SCope being slightly slower. iSEE-loom is memory efficient but there is a sudden increase in the preprocessing time at 250K and 500K cells. This is due to having eight panels in the default iSEE interface, each requiring a separate rendering of the visualization of the input cells. The defaults can be programmatically changed to having only one panel. This brings the preprocessing times down to under 2 min for 500K cells. Due to long preprocessing times we did not run iSEE-loom for datasets with more than 500K. Similarly, there is a sudden drop of loom-viewer efficiency at 2M cells. This effect was consistent across all five runs but we could not explain it. There is also a consistent drop in RAM usage by Single Cell Explorer at 50K cells, which we could not explain. In addition, we were not able to run SCope and Single Cell Explorer for datasets larger than 500K cells.

### Preprocessing time and memory

For four tools (iSEE-SCE, Single Cell Explorer, UCSC Cell Browser and cellxgene) the preprocessing memory and preprocessing time grow exponentially with the number of input cells. In case of iSEE-SCE and cellxgene this is due to the lack of HDF5 integration and loading of the full data in memory, which increases the server starting time. In contrast, the long preprocessing times of Single Cell Explorer and UCSC Cell Browser are explained by the required database and file preparation time, respectively. iSEE-SCE has the steepest RAM usage growth with the number of cells and failed to start for datasets larger than 50K cells. Among these four tools cellxgene has the shortest preprocessing times.

### User experience

In addition to the performance, we also compared the benchmarked tools by their user experience as shown in Table 2.

**Table 2.** UX features of the visualization tools

	cellxgene	iSEE	Loom-viewer	scSVA	SCope	Single Cell Explorer	UCSC Cell Browser
Ease of cell selection	✓	✓		✓	✓	✓	✓
Zoom in/out	✓	✓		✓	✓		✓
Multiple embeddings	✓	✓	✓		✓		✓
Highlight gene expression	✓	✓		✓	✓	✓	✓
Highlight metadata	✓	✓	✓	✓		✓	✓
Differential expression	✓	✓					
Cell-type annotation	✓			✓		✓	
Cell-type prediction						✓	
Web page loads fast	✓		✓	✓	✓	✓	✓

### Cell selection tools

For statistical analysis and comparison of groups of cells users need to select cell populations of interest. A majority of the tools provide selection functionality. The most flexible and user-friendly of them is the free-hand lasso selection, which is supported by cellxgene, SCope and Single Cell Explorer. It allows the user to select the cells by drawing a free shape curve around the cells of interest. A less flexible type of selection is rectangular selection, where the user is limited to drawing a rectangle around the cells of interest. It is supported by scSVA and UCSC Cell Browser. In addition, iSEE also supports a polygon version of the lasso selection, where the selection is made by pointing to the vertices of a polygon. To our knowledge, loom-viewer does not provide any method of selection.

### Zoom functionality

The ability to zoom in and out can be crucial to visually analyse and validate the data. Most of the tools have zooming functionality except loom-viewer and Single Cell Explorer. Similarly, the ability to switch between multiple embeddings (e.g. between tSNE and UMAP) can be very useful and help with the analysis. Again most of the tools have this functionality, except scSVA and Single Cell Explorer.

### Highlight by values

One of the most important features every single-cell visualization tool must have is the capability to highlight specific information. The user may want to highlight either gene expression levels (continuous scale) or cell metadata (usually on a discrete scale). Not surprisingly, this functionality is available in almost every tool with the exception of loom-viewer (for gene expression) and SCope (for cell metadata).

### Additional analysis

A useful feature of a visualization tool is the option of performing extra analysis on user-selected cells, such as e.g. cell-type annotation, differential expression analysis or marker gene identification. Three of the benchmarked tools (cellxgene, scSVA and Single Cell Explorer) have this functionality out of the box. iSEE supports this via custom panels.

### Page loading speed

Finally, an important indicator of a good user experience is the speed of web page loading. Once the back-end server is running most of the tools can serve the visualization to the user via a web page in a fast manner. One exception is iSEE. We tested it with the default setting of eight panels. At the moment the free version of the Shiny server does not support persistent R processes for faster load times (https://rstudio.com/products/shiny/shiny-server/) and therefore it starts a new R process for each new user, increasing the loading times and affecting the user experience when datasets are large.

## DISCUSSION

The size and volume of scRNAseq data has exponentially increased over the last decade and this has opened up new avenues of scientific discovery and understanding. There is a need among scientists to communicate their data to collaborators and colleagues for quick and easy exploration. The burden of computational resources and bioinformatics skills required to do this should ideally be removed from the recipients of the data.

Single-cell interactive analysis and visualization tools have been widely adopted by the research community. They make data import, public data access and analysis much easier for the users and accelerate the science. Furthermore, tools now exist (those with *Web Sharing* functionality in Table 1) that allow the user to host and share their scRNAseq data visualization with others on the web, due to recent advances in web technologies. These make it possible to share analysis results with others in a user-friendly manner, allowing for much faster scientific development. We believe that high complexity and dimensionality of scRNAseq data can only be revealed via comprehensive, interactive, and user-friendly tools that can provide shareable visualizations via the web. This is supported by the recent developments of scRNAseq visualization portals at large scientific institutes:

1. Single-Cell Expression Atlas (31) at the European Bioinformatics Institute - https://www.ebi.ac.uk/gxa/sc/home
2. Single-Cell Portal at the Broad Institute - https://singlecell.broadinstitute.org/single_cell
3. Cell Browser at the University of California Santa Cruz - https://cells.ucsc.edu/
4. Automated Single-cell Analysis Pipeline (ASAP) at École Polytechnique Fédérale de Lausanne - https://asap.epfl.ch/

To understand the current landscape of interactive analysis and visualization tools we compared (Table 1) several of the most popular based on their general qualities. Our results show that each tool has particular advantages and disadvantages and as such a simple ranking cannot be achieved. We looked specifically at the tools suitable for sharing an interactive visualization of results via a web interface (the *Web Sharing* row in Table 1). Again, in this case there is not one tool that stands out as significantly better than the rest in all categories. From our personal experience and partly supported by benchmarking, we currently recommend using cellxgene for publishing and sharing scRNAseq data.

Cellxgene performs well in terms of both memory and preprocessing times (Figure 1) and leads by the UX scores (Table 2). It also has a thriving community, is the most supported (Table 1) with 24 contributors and has the highest developer activity in the last 2 months. We have developed a detailed tutorial (https://cellgeni.readthedocs.io/en/latest/visualisations.html) for using cellxgene and how to convert data into the required input format.

Single-cell sequencing technologies are still in rapid development and we expect the dataset sizes (number of cells per dataset) to further grow in the next few years. Tools that use on-demand loading with linear or sublinear memory usage relative to cell count are best positioned to cope with this growth. Other tools will have to adapt and optimize in order to stay competitive. One way of optimization is to add integration with the HDF5 format as supported by our results in Figure 1. A simpler approach is to down-sample the data by selecting a small subset of cells in a random manner. Most of the tools support this functionality. When down-sampling it is important to make sure that rare cell populations are not removed. One such approach is geometric sketching (32) - a method to subsample massive scRNA-seq datasets while preserving rare cell states.

Additionally, there are several efforts to enrich visualizations by either using a 3D plot instead of 2D (18,33) or even by using virtual reality (34). The third dimension may allow resolution of cell-types not visible in two dimensions. Virtual reality can support multiple embeddings in the same VR space so that they can be directly compared.

This review represents a snapshot of a rapidly developing field and tools will catch up or drop out of contention and new tools will emerge. All of the tools in this review that are under active development are worth keeping an eye on. The designers and developers will need to not only think about efficiency and scalability of visualization but also about additional features that can enrich the data visualization and provide more scientific insights. An example is the user-friendly integration of a dataset under consideration with public scRNAseq data. This is already happening in commercial products, e.g. Bbrowser provides, for a selected group of cells, a suggestion of cell type based on publicly available data. It also provides the ability to search for specific cells from public data similar to the selected ones. It is worth noting that command line tools exist with exactly the same or similar functionality. However, putting this functionality into an interactive user-friendly interface allows sharing and exploration of results across the whole research community, facilitating scientific progress.
